# Mechanism of blueberry anthocyanins in ameliorating radiation-induced intestinal injury through gut microbiota modulation

**DOI:** 10.3389/fimmu.2026.1771482

**Published:** 2026-06-03

**Authors:** Aijing Yang, Beibei Zhang, Jiayuan Ju, Jinlong Tian, Dan Zhang, Jiabao Li, Lei Yang, Xi Zhang

**Affiliations:** 1 Department of Nutrition, General Hospital of Northern Theater Command, Shenyang, Liaoning, China; 2 School of Public Health, China Medical University, Shenyang, Liaoning, China; 3 College of Food Science, Shenyang Agricultural University, Shenyang, Liaoning, China; 4 School of Health & Intelligent Technology, Zhengzhou Health College, Zhengzhou, Henan, China

**Keywords:** radiation-induced intestinal injury, blueberry anthocyanins, gut microbiota, fecal microbiota transplantation, metabolomics

## Abstract

**Background:**

Radiation-induced intestinal injury denotes structural and functional impairment of the intestines resulting directly or indirectly from ionizing radiation, such as that employed in radiation therapy for malignant tumors in the abdominal or pelvic regions, within the treatment area. It is a prevalent and severe consequence of radiation therapy for abdominal and pelvic tumors, greatly affecting treatment outcomes and patients’ quality of life. Western medicine primarily employs symptomatic management, including medical drug therapy, hyperbaric oxygen therapy, nutritional support therapy, endoscopic and surgical interventions, as well as stem cell transplantation. However, current therapeutic agents for RIII fall far short of achieving ideal outcomes. Previous studies indicate that blueberry anthocyanins (BA) possess not only anticancer properties but also potent anti-inflammatory, antioxidant, and anti-radiation activities.

**Methods:**

Seventy male C57BL/6J mice (6–8 weeks old) were categorized into five groups: a normal control group (Con), an irradiation-only group (IR), groups administered low-dose BA prior to and following irradiation (IR+BA-L, 100 mg/kg bw), a high-dose BA group (IR+BA-H, 200 mg/kg bw), and a group receiving the clinical radiation protection agent amphotericin (IR+WR-2721, 30 mg/kg bw). Furthermore, a group for antibiotic-induced gut microbiota depletion and a validation group for fecal microbiota transplantation (FMT) were established. A model of radiation-induced intestinal damage was created utilizing a single 14 Gy dose of abdominal X-ray irradiation. The irradiation field encompassed a 2.5 cm by 20 cm region extending from the pubic symphysis to the xiphoid process. BA was supplied through oral gavage from 14 days prior to irradiation until 3.5 days post-irradiation at a dose of 0.2 mL/day per mouse, whereas the normal control group received saline via gavage for 14 consecutive days. Tissue samples were obtained 3.5 days post-irradiation. Through the assessment of survival rates, body mass, intestinal histopathology, inflammatory cytokine concentrations (IL-1β, IL-6, TNF-α), oxidative stress indicators (SOD, MDA), and intestinal barrier proteins (occludin, claudin-1), alongside immunohistochemical analysis of the proliferative cell marker Ki67 and the Paneth cell marker lysozyme, the proliferation and differentiation of intestinal stem cells will be evaluated. Furthermore, 16S rDNA sequencing was executed on colonic contents, and non-targeted metabolomics analysis was undertaken on colonic tissue to assess alterations in gut microbiota and host metabolism.

**Results:**

Mice treated with BA intervention exhibited significant alleviation of intestinal injury, manifested as reduced weight loss, increased survival rate, markedly elongated colon length, substantially mitigated intestinal damage, and elevated histological scores. Concurrently, BA therapy downregulated levels of pro-inflammatory cytokines IL-1β, TNF-α, and IL-6, while decreasing elevated SOD content and MDA levels. Immunohistochemical analysis revealed that BA intervention significantly increased Ki67^+^proliferating cells and Lysozyme^+^ Paneth cells in the crypt region of the colon. Western blot analysis demonstrated that BA upregulated the levels of intestinal tight junction proteins Occludin and Claudin-1. 16S rDNA sequencing revealed that BA improved gut microbiota composition by increasing the abundance of beneficial bacteria and reducing pathogenic bacteria. Metabolomic analysis revealed altered metabolic patterns in mouse colon tissue following both radiation exposure and BA intervention. BA restored levels of 53 potential biomarkers. Finally, fecal microbiota transplantation (FMT) validated BA’s protective effect against RIII via the gut microbiota.

**Conclusions:**

The results of this study show that blueberry anthocyanin (BA) exerts significant protective effects against radiation-induced intestinal injury (RIII). It ameliorates RIII by modulating gut microbiota composition and associated metabolites. FMT experiments further validate that BA’s protective action depends on gut microbiota regulation. The findings suggest that BA, as a gut microbiota-modulating agent, holds promise for preventing and treating RIII.

## 1 Introduction

With the rapid advancement of nuclear energy and technology, radionuclides have found extensive applications across multiple sectors, including national defense, industry, agriculture, communications, transportation, environmental protection, and resource development. They also play a pivotal role in nuclear medicine diagnostics, therapy, and life science research, progressively increasing human exposure to radiation environments (1). Ionizing radiation (IR) can directly or indirectly cause ionization in matter. Its sources include alpha rays, beta rays, gamma rays, and high-speed neutrons, electrons, and protons (2). In daily life, humans are inevitably exposed to various natural and artificial radiation sources. Prolonged radiation exposure significantly impacts hematopoietic function, the digestive system, and the immune system through mechanisms such as microcirculatory disorders, apoptosis, carcinogenesis, and DNA mutations (3).

Radiation-induced bowel damage, or radiation enteritis, is a deleterious response in the intestines elicited by therapeutic ionizing radiation applied to the abdominal or pelvic area. It is considered a dose-limiting adverse effect of cancer radiation. Pathologically, it is defined by injury to the intestinal mucosa and its milieu due to radiation energy, presenting as a continuous pathological development from acute inflammatory responses to chronic fibrotic lesions (4). Radiation-induced intestine damage is a prevalent consequence of radiation, especially in the treatment of abdominal tumors. Exposure to radiation in intestinal organs results in the onset of radiation enteritis. Despite developments in contemporary radiotherapy treatments that have markedly diminished the occurrence and severity of radiation-induced intestinal damage, certain patients continue to endure various levels of injury (5). Furthermore, radiotherapy can cause severe imbalances in the intestinal microbiota, affecting patient prognosis. Disrupted gut flora may lead to overgrowth of opportunistic pathogens. These pathogens can penetrate the damaged intestinal mucosal barrier, triggering serious complications such as enteric infections, endotoxemia, and sepsis (6).

To mitigate IR damage to the human body, the development of radiation protective agents has become a research priority. Amifostine (WR-2721) is the sole radiation protection agent licensed by the U.S. FDA and is regarded as the most authoritative positive control medicine in the domain. This study utilizes it as the gold standard positive control medication to validate the efficacy of the radiation model; however, its practical application is constrained by considerable hazardous side effects (7). In contrast, natural plant extracts exhibit low toxicity and broad-spectrum effects, capable of influencing multiple organs and targets. Consequently, the development of highly effective, low-toxicity natural radiation protection agents has become a major focus (8). In preclinical murine research concerning whole-body or abdominal radiation protection, 30 mg/kg body weight is one of the most often utilized and proven efficacious doses of WR-2721, delivered either by intraperitoneal injection or oral gavage. This dose offers substantial survival advantages and tissue safeguarding before radiation exposure and has been recognized as the standard positive control dose in various high-quality investigations. This study used the clinically licensed radiation protective drug amphotericin (WR-2721) as a positive control to assess whether blueberry anthocyanins exhibit radiation protective properties comparable to or beyond those of amphotericin.

Anthocyanins are a class of natural compounds widely distributed in dark-colored fruits and vegetables such as blueberries, raspberries, purple cabbage, and purple sweet potatoes. The fundamental structure comprises a flavanone cation. Anthocyanins can be categorized into acylated and non-acylated forms based on the presence of an acyl group in their chemical structure, with acylated anthocyanins demonstrating enhanced stability and further classed according to the quantity of acyl groups present. Acylated anthocyanins possess one or more acid groups ester-linked to the sugar moiety, and they can be categorized as monoacylated, diacylated, triacylated, and tetraacylated forms according to the quantity of acyl groups present. pH, light exposure, temperature, and their intrinsic structure influence the hue and stability of anthocyanins. For instance, they have a red hue under acidic conditions and progressively transition to blue as the pH rises (9). Research has confirmed that anthocyanins possess multiple physiological functions, including free radical scavenging, lipid-lowering, and antitumor effects. Notably, their exceptional free radical scavenging properties enable effective defense against radiation damage, thereby producing radioprotective effects (10). However, studies on the protective effects of blueberry anthocyanins (BA) against RIII have yet to be identified. The doses of blueberry anthocyanins (BA) employed in this investigation (100 and 200 mg/kg bw) were determined based on recognized efficacy and safety profiles. Prior rodent studies have consistently shown that oral treatment of BA within the 50–200 mg per kilogram of body weight produces strong anti-inflammatory and antioxidant benefits across several disease models without considerable toxicity (11). Our initial trials demonstrated that these doses are well-tolerated and provoke a quantifiable protective response against radiation-induced intestinal damage, with 200 mg/kg of body weight finally recognized as the best intervention dose.

As one of the fastest-dividing tissues in the human body, small intestinal tissue is particularly susceptible to IR effects. Intestinal cells, especially epithelial cells, become primary target organs for radiation damage due to their rapid proliferation and renewal characteristics (12). In recent years, cancer incidence has significantly increased. Radiotherapy, an effective treatment for pelvic and abdominal malignancies, is one of the primary causes of radiation-induced bowel injury (RIII) (13).

Fecal microbiota transplantation (FMT) involves transferring the gut microbiota from a healthy donor into a patient to introduce or restore a balanced microbial community in the gut. It has become an effective treatment for recurrent Clostridium difficile infection (CDI) and is included in clinical guidelines (14). It also demonstrates therapeutic potential in treating microbe-disruption-related conditions such as irritable bowel syndrome, constipation, autism, liver disease, radiation enteritis, graft-versus-host disease, tumors, and tumor-associated complications (15). Research indicates that successful FMT is marked by the colonization and proliferation of specific beneficial bacteria (e.g., short-chain fatty acid-producing bacteria) from the donor in the recipient’s gut, thereby restoring microbial diversity, suppressing pathogenic bacteria, and alleviating intestinal inflammation (16).

In this study, to investigate the preventive and therapeutic effects of BA on RIII and its potential mechanisms, an X-ray-induced RIII mouse model was established. The study examined the effects of BA intervention on mice exposed to 14 Gy abdominal irradiation, focusing on general vital signs, histopathology, inflammatory factors, oxidative stress, and intestinal barrier function. We comprehensively applied experimental techniques, including colonic metabolomics analysis and 16S rDNA high-throughput sequencing, to explore the protective effects of BA against RIII and its potential mechanisms. This not only provides a reference for developing natural radiation protective agents but also offers important support for enhancing public health protection measures in radioactive environments.

## 2 Materials and methods

### 2.1 Animals experiments

Seventy healthy 6–8 week-old male C57BL/6 SPF-grade mice weighing approximately 20–25 g were procured from Beijing Huafukang Biotechnology Co., Ltd. The mice were first separated into individual cages and then acclimated for one week before experimentation. All mice were housed uniformly in an SPF-grade animal facility with a light-dark cycle. with room temperature maintained at (23 ± 1.5)°C and relative humidity between 60% ± 10%. They were fed standard rodent chow with unrestricted access to food and water. All animal experimental supplies, including standard rodent feed, water, bedding, and other materials, complied with the relevant regulations of the Animal Ethics Committee of Shenyang Agricultural University. Animal Experiment License No. 24051501.

### 2.2 A model of radiation-induced intestinal damage

Male C57BL/6J mice, aged 6 to 8 weeks, were initially acclimatized to a regular diet for one week. Following the assessment of 70 mice, the donor cohort was partitioned into five subgroups (n=8 per group): the control group (Con), the irradiation group (IR), the low-dose anthocyanin group (IR+BA-L; 100 mg/kg bw), the high-dose anthocyanin group (IR+BA-H; 200 mg/kg bw), and the aminoplatin group (IR+WR-2721; 30 mg/kg bw). BA, in conjunction with saline, was supplied orally to the mice via gavage at a dose of 0.2 mL/day per mouse from 14 days prior to irradiation until 3.5 days post-irradiation. The control and the IR groups were administered daily saline gavage for 14 consecutive days. The FMT cohort was categorized into three groups: the irradiation group combined with the normal microbiota transplantation group (IR+FMT-Con), the irradiation group combined with the irradiated microbiota transplantation group (IR+FMT-IR), and the irradiation group combined with high-dose blueberry anthocyanin intervention followed by microbiota transplantation group (IR+FMT-BA), with n = 8 mice in each group. The microbiota transferred to the recipient groups were Con, IR, and IR+BA-H, respectively, aligning with the donor groups. To create a pseudo-germ-free mouse model, the FMT group was initially given a four-drug broad-spectrum antibiotic cocktail combined with sterile water for a duration of 5 days. The formulation, comprising 1 g/L ampicillin, 1 g/L metronidazole, 1 g/L neomycin sulfate, and 0.5 g/L vancomycin, was provided daily by drinking water. A 5-day gavage intervention ensued, utilizing a solution comprising 100 mg of ampicillin, 100 mg of metronidazole, 100 mg of neomycin sulfate, and 50 mg of vancomycin dissolved in 4 mL of PBS to formulate an antibiotic solution, which was administered to the mice at a dose of 0.2 mL/day. The sterile fecal filtrate obtained from donor mice following modeling and tissue collection was administered to recipient mice at a dose of 0.2 mL per day. FMT transplantation was conducted daily from 10 days prior to irradiation until 3.5 days after irradiation. Mice were anesthetized 20 minutes before irradiation. Post-anesthesia, they were placed supine on a fixation board and administered a singular abdomen X-ray dose of 14 Gy via a Swiss Elekta Precise linear accelerator (6 MV). The irradiation field spanned from the xiphoid process to the pubic symphysis, while the other regions were protected by lead plates. The control group of mice was administered 0 Gy of radiation. The irradiated group received a dose rate of 200 cGy/min from a source positioned 100 cm from the skin, with an irradiation field measuring 2.5 cm in length and 20 cm in width. Samples were obtained 3.5 days post-irradiation.

### 2.3 Histopathology

After being preserved in 4% paraformaldehyde for 24 to 72 hours, colon tissues were rinsed with PBS for 30 minutes. Sequential dehydration was carried out in 70% ethanol for one hour, 85% ethanol for one hour, 95% ethanol for one hour, 100% ethanol I for thirty minutes, and 100% ethanol II for one hour. Tissues were then cleaned for 30 minutes in xylene I and 50 minutes in xylene II. Tissues were submerged in paraffin I for an hour and paraffin II for a whole night in order to achieve paraffin penetration. The infiltrating tissue block was positioned in the middle of a mold, filled with molten paraffin, and quickly cooled on an ice bath to complete the embedding process. The paraffin block was taken out of the mold once it had solidified. A rotary microtome was used to cut slices of 5 μm thickness after the paraffin block was trimmed to the required shape. After allowing the sections to fully expand in clean water at 40 °C to 45 °C, they were lifted up onto adhesive-coated slides, labeled one after the other, and baked for several hours at 60 °C to produce paraffin sections that were ready for further histological staining.

### 2.4 Immunohistochemistry

Immunohistochemistry (IHC) for lysozyme and Ki67 was conducted on paraffin-embedded slices of colon tissue. Following deparaffinization, rehydration, and antigen retrieval in EDTA buffer (pH 8.0), endogenous peroxidase activity was inhibited using 3% H_2_O_2_. Sections were subsequently blocked with 3% BSA and incubated overnight at 4 °C with the primary antibodies: anti-Lysozyme (1:1000, #A10972) and anti-Ki67 (1:500, #GB111141). Following washing, slices were incubated with HRP-conjugated goat anti-rabbit IgG (1:100, #AS014) for 50 minutes at ambient temperature, succeeded by DAB chromogenic development and hematoxylin counterstaining. The Histochemical Score (H-score) was utilized to evaluate staining, conducted by two blinded observers who scored staining intensity (0–3) and the percentage of positive cells in five randomly selected high-power fields (200×) per sample.

### 2.5 Enzyme-linked immunosorbent assay

Concentrations of TNF-α, IL-6, and IL-1β in mouse colon tissue homogenates were quantified utilizing commercial ELISA kits in accordance with the manufacturer’s guidelines. Remove intestinal tissue from the -80 °C freezer and add pre-chilled PBS at a 1:10 mass-to-volume ratio. Centrifuge the homogenate and collect the supernatant as the test sample. Detect TNF-α, IL-6, and IL-1β levels in mouse intestinal tissue using an ELISA kit. Measure absorbance at 450 nm using an ELISA reader. Calculate inflammatory cytokine levels (units: pg/ml) in each group of mouse tissues via standard curve analysis.

### 2.6 Oxidative stress

Take 50 μL of serum and place it on ice at 4 °C for later use. Set up the assay system according to the Nanjing Jiancheng SOD Assay Kit instructions, add the corresponding reagents, and measure the absorbance at 550 nm using a microplate reader. Calculate the SOD level following the method described in the instructions. Detect using the Biyuntian MDA Assay Kit, measure the absorbance at 532 nm, and calculate the MDA content according to the instructions.

### 2.7 Western blot

Western blot analysis was conducted to assess the expression of tight junction proteins (occludin and claudin-1) in colonic tissue. Proteins were extracted via RIPA lysis buffer and quantified via a BCA kit, and equal quantities (20 µg) were subjected to SDS-PAGE before being transferred to PVDF membranes. Membranes were obstructed using 5% non-fat milk-TBST and subsequently incubated overnight at 4 °C with primary antibodies: anti-occludin (1:1000, #ET1701-76, HuaAn), anti-claudin-1 (1:1000, #HA721999, HuaAn), and anti-β-actin (1:1000, #HA7222023, HuaAn) serving as the loading control. Following washing, membranes were incubated with HRP-conjugated goat anti-rabbit IgG (1:5000, #HA724235H, HuaAn) for one hour at ambient temperature. Protein bands were identified by enhanced chemiluminescence (ECL) and quantified utilizing ImageJ software, with target protein levels standardized to β-actin.

### 2.8 Intestinal contents 16S rDNA sequencing

Colon tissue samples from the donor groups (Con, IR, and IR+BA-H groups; n = 6 per group) were analyzed using 16S rDNA sequencing. DNA extraction and 16S rDNA sequencing: DNA was extracted from mouse colonic contents following the CTAB kit protocol (17), with electrophoresis used to assess quality and concentration. DNA was diluted to 1 ng/μL and amplified using barcoded primers targeting the V4 region of 16S rDNA. PCR products were pooled at equal concentrations, purified via 2% agarose gel electrophoresis, and target bands recovered using a Qiagen kit. Libraries were constructed using the TruSeq^®^ DNA PCR-Free Sample Preparation Kit. Quantification was performed via Qubit, Agilent Bioanalyzer 2100 System, and Q-PCR. Sequencing was conducted using the NovaSeq 6000. Intestinal contents 16S rDNA sequencing was performed by Wuhan Maiwei Metabolic Biotechnology Co., Ltd.

### 2.9 Non-targeted metabolomics

Colon tissue specimens from the donor groups (Con, IR, and IR+BA-H; n = 6 per group) underwent untargeted metabolomic analysis. To assess instrument stability, quality control (QC) samples were generated by combining equal aliquots of all experimental samples and analyzed at predetermined intervals (every 10 injections) during the LC-MS analytical run. Mass spectrometry data were converted to mzXML format using ProteoWizard and processed with XCMS (including peak extraction, alignment, and time correction). Data preprocessing included: (1) removal of peaks with missing rates exceeding 50% across all samples; (2) imputation of the remaining missing values using the k-nearest neighbors (KNN) algorithm (k=10) in the R package impute; (3) correction of peak areas using support vector regression (SVR). Metabolite identification utilized laboratory databases, public databases, prediction databases, and the metDNA method (18). Compounds with composite scores>0.5 and QC sample CV<0.3 were extracted and merged across positive/negative ion modes (retaining the compound with the highest confidence level and lowest CV). Unit variance (UV) scaling was applied using R’s prcompfunction (scale=TRUE), followed by principal component analysis (PCA) for sample distribution and stability assessment. Subsequently, orthogonal partial least squares-discriminant analysis (OPLS-DA) was performed using the MetaboAnalystR package. Differentially expressed metabolites with variable importance in projection (VIP)>1 and *P*<0.05 (Student’s t-test) were selected. Metabolite concentration data underwent UV scaling, followed by heatmap visualization and hierarchical clustering using the ComplexHeatmap package. Finally, metabolic pathway enrichment analysis was conducted based on the Kyoto Encyclopedia of Genes and Genomes (KEGG) database.

### 2.10 Fecal microbiota transplantation protocol

To create a pseudo-germ-free mouse model, the mice underwent a 5-day treatment with a quadruple broad-spectrum antibiotic cocktail in sterile water. The triple antibiotic solution, comprising 1 g/L of ampicillin, 1 g/L of metronidazole, 1 g/L of neomycin sulfate, and 0.5 g/L of vancomycin, was delivered daily by drinking water. A 5-day gavage intervention ensued, utilizing a solution of 100 mg of ampicillin, 100 mg of metronidazole, 100 mg of neomycin sulfate, and 50 mg of vancomycin, all dissolved in 4 mL of PBS, which was delivered daily via gavage to the mice. With the exception of the normal control group, mice in the remaining groups were administered 0.2 mL of this solution via gastric lavage daily for five consecutive days. Throughout this five-day interval, the concentration of the sterile antibiotic solution ingested by the mice was reduced by fifty per cent, and it was made again each day. Following a 3-day repose to facilitate microbial colonization (19), The efficacy of microbial clearance was validated by 16S rDNA analysis. Intestinal contents were obtained from donor mice 3.5 days post-irradiation. Subsequent to processing, they were delivered through gavage to the designated recipient mice. The intestinal contents from each group were diluted with PBS at a ratio of 100 mg to 1 mL, meticulously mixed with a disposable sterile mixer, filtered through a sterile cell strainer to exclude big particles, and subsequently transferred to 50 mL sterile centrifuge tubes. Centrifuge at 6000 revolutions per minute for 15 minutes at 4 degrees Celsius. Isolate the supernatant, combine it with glycerol in a 1:1 ratio, and preserve at -80 °C. The filtered fecal filtrate was distributed into sterile 1.5 mL centrifuge tubes to enable use and prevent repeated freezing. With the exception of the normal control group, fecal microbiota transplantation (FMT) was administered daily from 10 days prior to irradiation until 3.5 days post-irradiation, at a dose of 0.2 mL per mouse. The irradiation protocol aligned with that of the donor group.

### 2.11 Statistical analysis

The donor experiment data were analyzed utilizing SPSS 22.0 software, with results shown as mean ± standard error of the mean (SEM). Variations in means across several groups were evaluated using one-way analysis of variance (ANOVA), with pairwise comparisons conducted via the LSD test or Dunnett’s t-test where applicable. Statistical analysis for non-normally distributed data was conducted using the rank-sum test. Statistical graphs contrasting the antibiotic clearance group with the receiving group were produced utilizing GraphPad Prism 9.5 software. Quantitative data are typically expressed as mean ± standard deviation (mean ± SD). Normally distributed variables were assessed by one-way analysis of variance (ANOVA), whereas non-normally distributed variables were examined using the Kruskal-Wallis test. The threshold for statistical significance was established at *p* < 0.05.

## 3 Results

### 3.1 BA administration mitigates RIII

**Figure 1:**
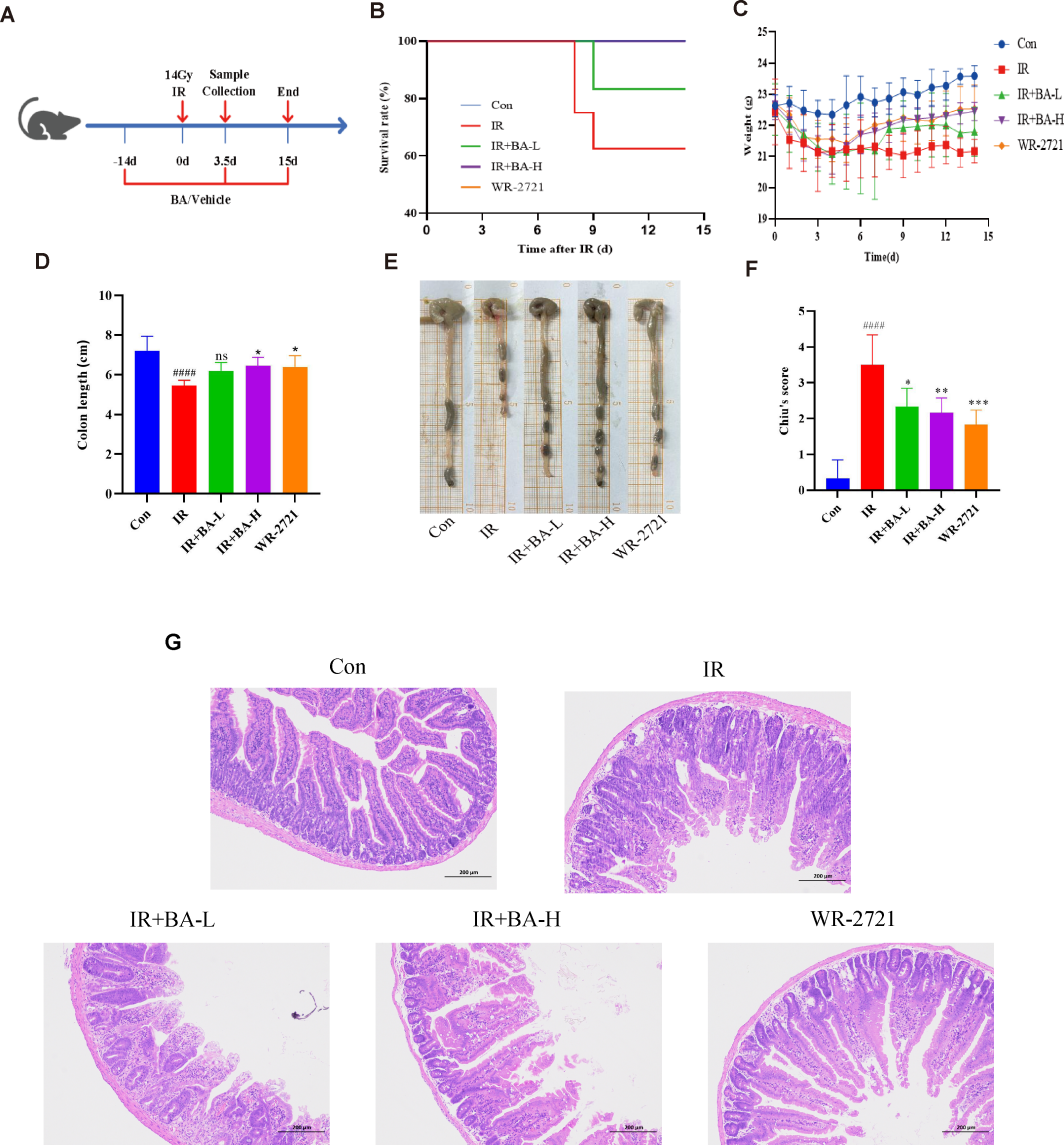
Effects of BA on survival, body weight, and intestinal injury in RIII mice (Donor group ‘WR-2721’ denotes the group that received WR-2721 intervention following irradiation IR+WR-2721). **(A)** Schematic of RIII modeling, intervention, and tissue sampling; **(B)** Survival analysis curve for RIII mice(n=8); **(C)** Body weight curve of RIII mice(n=8); **(D)** Quantitative Analysis of Colon Length in Different Groups of RIII Mice(n=4), compared with the Con group, ^#^*P* < 0.05; Compared with the IR group, *P < 0.05, **P < 0.01, ***P < 0.001; **(E)** Colonic length in each group of RIII mice; **(F)** Histopathological injury scoring of RIII mouse intestinal tissues(n=6); **(G)** Image of HE-stained intestinal tissue from RIII mice. Body weight changes of mice in each group after irradiation. Data are presented as the mean body weight of surviving mice at each time point. The decrease in group size over time, particularly in the IR group, corresponds to the mortality data shown in **(B)**.”.

Flowchart of the Experimental Protocol for Radiation-Induced Intestinal Injury (**Figure 1A**). IR mice showed lower food and water intake, arched backs, sluggish reflexes, trembling fur with poor luster, and lethargy in comparison to the Con group. Additionally, they showed signs of intestinal distress, such as watery feces, which validated that the RIII model had been successfully established. All of the BA and WR-2721 groups remained healthy in comparison to the IR group, with consistent food and drink consumption and mild loose stools. This suggests that BA can improve irradiated mice’s survival status to some extent. Additionally, the survival of mice in the IR group was significantly reduced following abdominal irradiation with a 14 Gy dose. The BA injection dramatically raised the mice’s survival rate when compared to the IR group. The survival duration of RIII mice was considerably extended by BA intervention, while animals in the IR group started to die on day 8 (**Figure 1B**). Additionally, following radiation, body weight variations in all groups—aside from the blank group—showed varied degrees of loss, peaking on day 4. While the IR group showed no discernible change, the IR+WR-2721 group displayed the slowest fall, followed by a progressive recovery in the IR+BA-L and IR+BA-H groups (**Figure 1C**). These findings show that BA has protective effects on RIII mice and reverses the post-radiation weight loss trend in mice. Nonetheless, in analyzing the alterations in body weight, we observed that the average body weight in the IR group throughout the later phases was determined based on the surviving mice, potentially introducing a survival bias. Nonetheless, the fundamental conclusion of this study—the radioprotective action of blueberry anthocyanins (BA)—remains unchanged by this. IR+BA-H and IR+WR-2721 groups demonstrated uniform weight recovery beginning on days 4 to 5 post-irradiation, with survival rates in these groups at 100%. This suggests that weight recovery signifies a true protective effect of BA on all participants, rather than a statistical anomaly, so robustly supporting the conclusion that BA can mitigate radiation-induced cachexia. Additionally, Following the BA and WR-2721 interventions, colon length showed some recovery (*P* < 0.05), although the IR group’s colon length was much shorter than the Con group’s (**Figures 1D, E**). Concurrently, IR causes pathological alterations such as mucosal structural damage, crypt atrophy, reduction in crypt cells, and destruction of small intestinal villi structure (20). Histopathological changes were investigated using HE staining in order to assess BA’s protective effect on RIII mice in more detail. There were no indications of damage or other pathological changes in the Con group’s intestinal architecture, which included well-organized and comparatively complete villi, a large number of glands, minor intestinal inflammation, and crypt outlines that were easily visible inside the mucosal layer. The IR group showed reduced crypt numbers, extensive inflammatory cell infiltration, altered crypt architecture, and severely shortened and lost villi 3.5 days after irradiation (**Figure 1G**). Increased crypt depth and more complete villus organization were two notable improvements in intestinal structure brought about by BA and WR-2721 therapies. Chiu’s pathological damage rating system was used to evaluate intestinal damage in irradiated mice. Following irradiation, intestinal damage scores considerably increased; however, following BA and WR-2721 intervention, they dramatically decreased, especially in the IR+BA-H and IR+WR-2721 groups (*P* < 0.01) (**Figure 1F**). In conclusion, BA intervention reduces intestinal structural damage caused by radiation.

### 3.2 BA alleviates RIII in a gut microbiota-dependent manner

**Figure 2:**
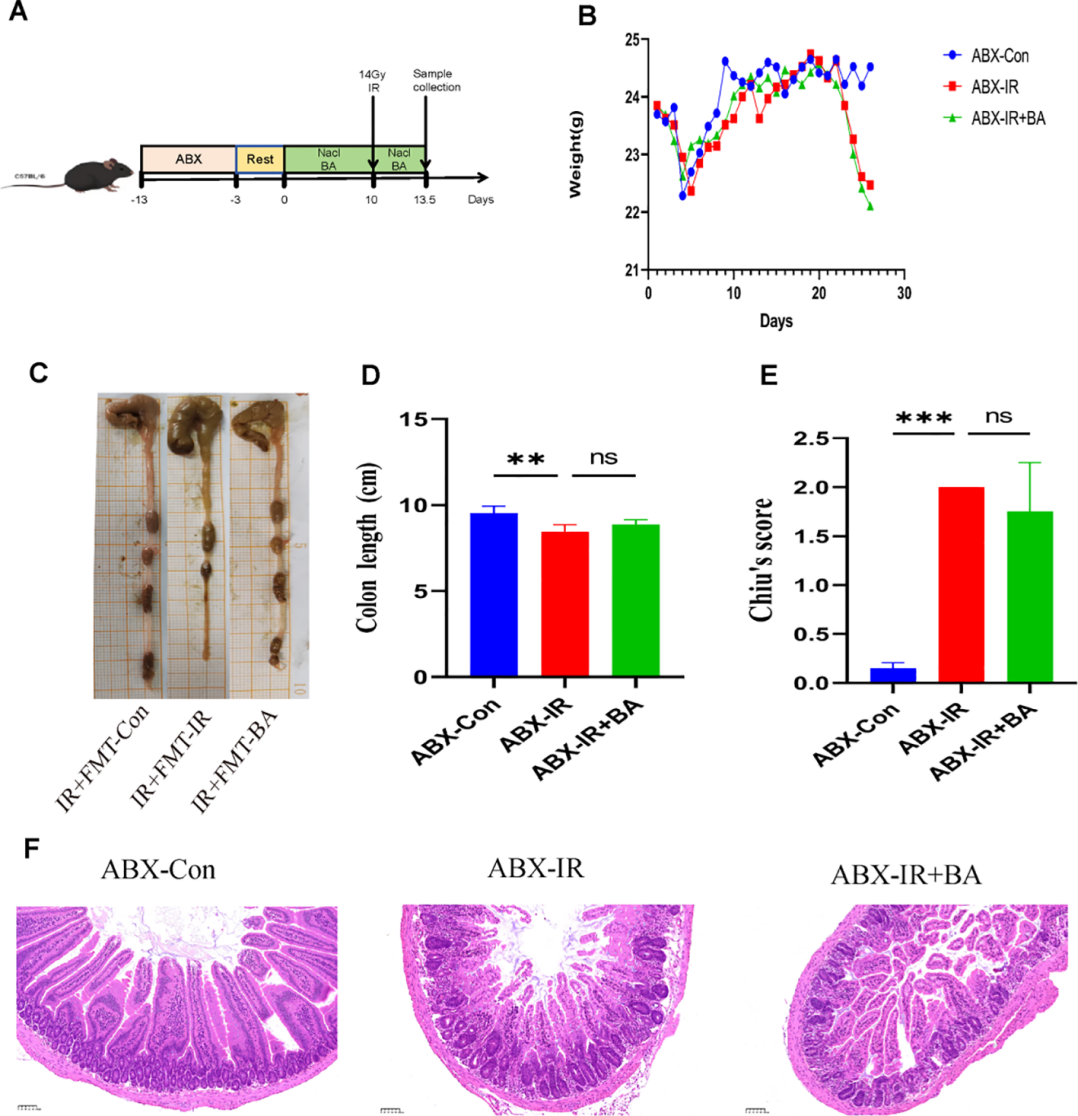
BA alleviates RIII in a gut microbiota-dependent manner (antibiotic clearance group). **(A)** Schematic of FMT modeling, intervention, and sampling; **(B)** Body weight changes in mice across groups (n=8); **(C)** Colon length in each group (n=4); **(D)** Quantified colon length in mice across groups (n=4), **P<0.01, ***P<0.001; **(E)** Histopathological injury scores in intestinal tissue across groups (n=4); **(F)** Representative HE-stained intestinal tissue sections from each group.

Experimental Flowchart for Pseudogastrectomy in Mice (**Figure 2A**): After generating pseudo-germ-free mice, we observed a decrease in body weight across all groups, followed by a subsequent recovery (**Figure 2B**). All groups showed no discernible increases in colon length (**Figure 2C**). At the same time, there were no significant changes in colon length measurements following BA intervention(**Figure 2D**). When compared to the control group, HE staining showed varying degrees of intestinal damage in all irradiation groups, including substantial inflammatory cell infiltration, atrophy, disturbed villus organization, and disruption of the crypt structure. Nevertheless, no notable alterations were brought about by the BA intervention (**Figures 2E, F**). These results suggest that the gut microbiota is necessary for BA’s anti-inflammatory protective action against colonic inflammation.

### 3.3 FMT improves RIII

**Figure 3:**
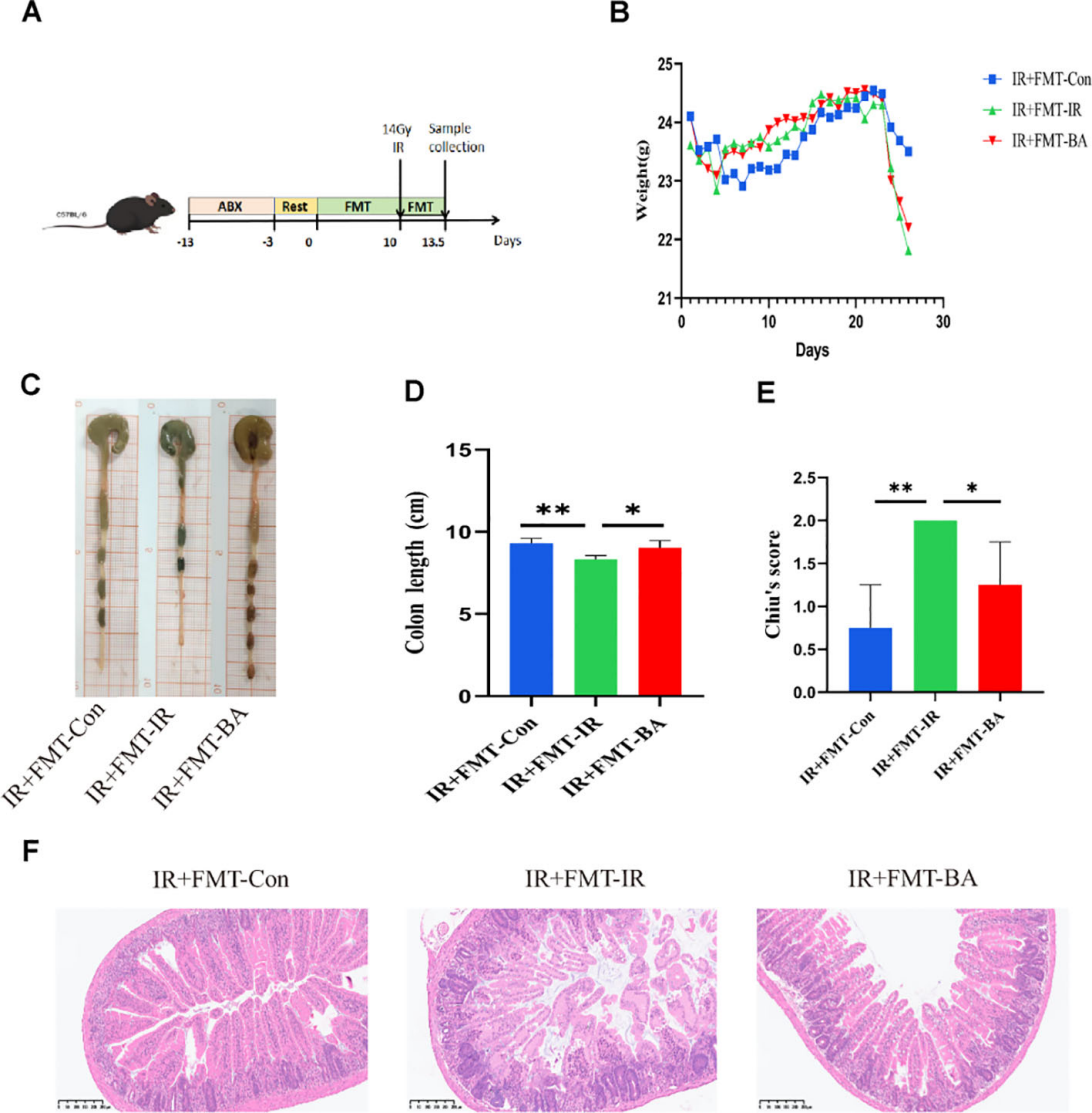
Effects of FMT on survival, body weight, and intestinal injury in RIII mice (recipient group). **(A)** Schematic of FMT modeling, intervention, and tissue sampling; **(B)** Body weight changes in each group (n=8); **(C)** Quantitative analysis of colon length in each group (n=4), *P < 0.05, **P < 0.01 **(D)** Colon length in each group (n=4); **(E)** Histopathological injury scores in intestinal tissue across groups (n=4); **(F)** Representative HE-stained intestinal tissue images from each group.

BA Fecal Microbiota Transplantation Flowchart (**Figure 3A**). After FMT intervention no mouse deaths were noted in any of the groups. Weight curves revealed that all other groups—aside from the Con group—showed weight decrease after antibiotic therapy, peaking between days 5 and 7 before progressively rising. All groups lost weight to varied degrees after irradiation, with day 4 post-irradiation showing the lowest amount (**Figure 3B**). All groups also showed colon shortening after radiation, along with varied degrees of cecal hypertrophy, in comparison to the control group. The colon length was shortest in the IR+FMT-IR group. Following the intervention, both the IR+FMT-BA and IR+FMT-Con groups showed a significant recovery in colon length compared to the IR+FMT-IR group (P<0.05). This indicates that transplantation of either normal microbiota or BA-modulated microbiota can effectively mitigate radiation-induced colon shortening.(*P* < 0.05) (**Figures 3C, D**). Following 14 Gy whole-body irradiation, histological analysis of the jejunal structure showed that all irradiated groups had different levels of intestinal damage as compared to the Con group. Significant intestinal gland dysplasia, strongly stained cellular atypia, widespread inflammatory cell infiltration, disordered villus arrangement with atrophy, and crypt structural degradation were all observed in the IR+FMT-IR group. The IR+FMT-BA group, on the other hand, showed significantly fewer pathological alterations, including well-organized villi, minimal inflammatory cell infiltration, no substantial atrophy or ulceration, and a lower intestinal injury score (*P* < 0.05) (**Figures 3E, F**). The results show that radiation-induced intestinal damage is lessened by the transplanting of both normal and anthocyanin-treated microbiota, indicating a protective impact on the gut.

### 3.4 BA alleviates inflammation and oxidative stress in RIII mice

**Figure 4:**
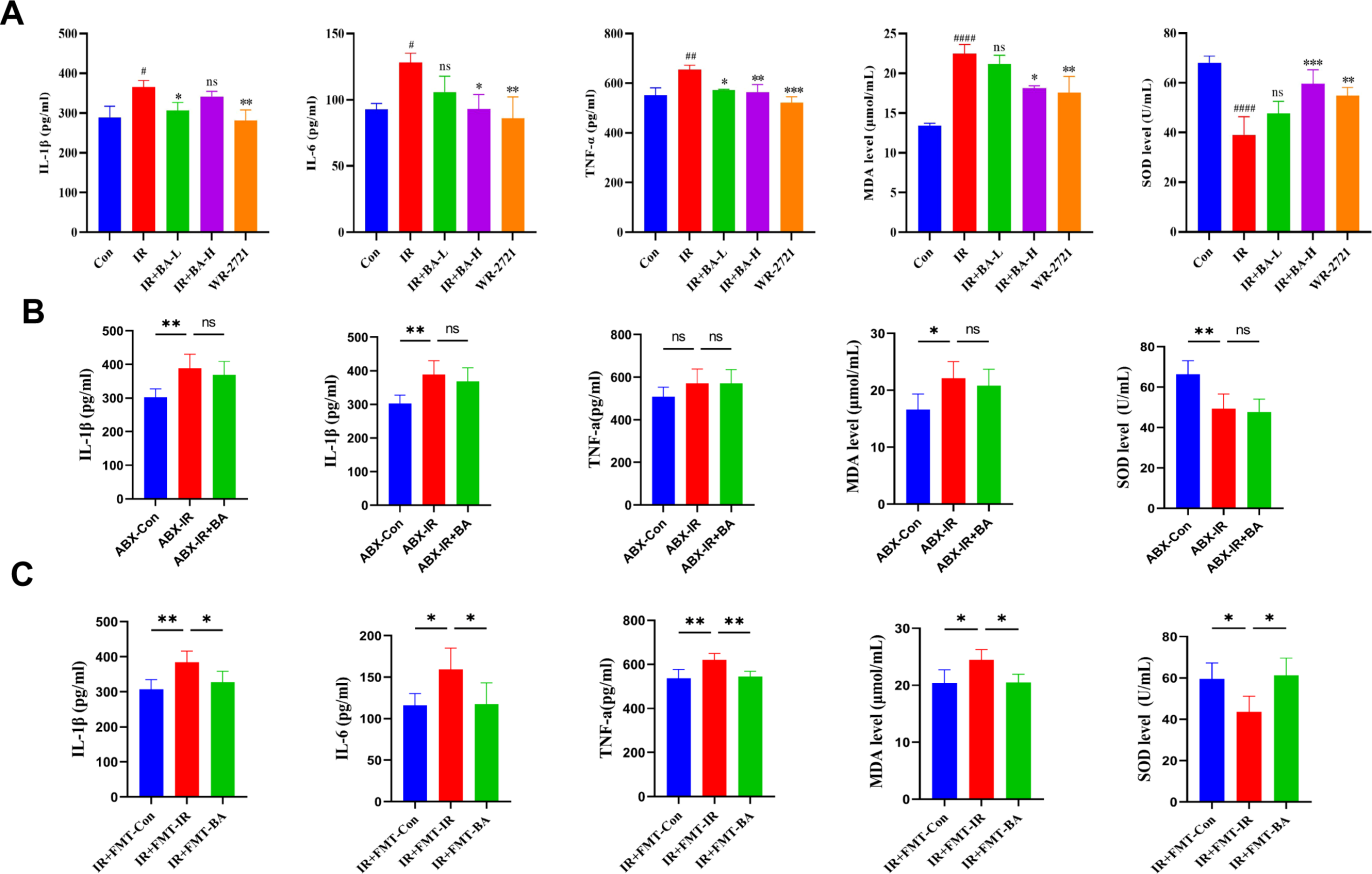
Effects of BA on inflammation and oxidative stress in RIII mice. **(A)** Levels of IL-1β, IL-6, TNF-α in intestinal tissue and MDA and SOD in serum of donor mice (n=3); Compared with the Con group, *^#^P* < 0.05, *^##^P* < 0.01, ####*P*<0.0001; Compared with the IR group, *P < 0.05, **P<0.01, ***P<0.001; ns indicates no statistically significant difference. (Donor group) **(B)** Levels of inflammatory factors and oxidative stress markers in antibiotic-cleared mice (n=5); *P<0.05, **P<0.01, ns indicates no statistically significant difference. (Antibiotic Clearance Group) **(C)** Inflammatory factors and oxidative stress levels in FMT mice (n=5); *P<0.05, **P<0.01.(recipient group).

According to research, ROS produced by radiation trigger a number of inflammatory and immunological reactions (21), and proinflammatory cytokine production is essential for RIII (22). Thus, we assessed intestinal tissue levels of proinflammatory cytokines TNF-α, IL-6, and IL-1β, as well as colon length in mice. The intestinal tissues of the IR group exhibited noticeably higher expression of these inflammatory factors. TNF-α expression was decreased in mice treated with BA at different doses with WR-2721. Furthermore, BA dramatically reduced radiation-induced expression of the pro-inflammatory cytokine TNF-α, as seen by the suppression of TNF-α levels by BA groups that was similar to that seen in the WR-2721 group (*P* < 0.01). BA-H and WR-2721 therapy significantly decreased IL-6 content (*P* < 0.05) in comparison to the IR group. When compared to the IR group, the BA-L group did exhibit a trend toward lower IL-6 levels, but this difference was not statistically significant. The WR-2721 intervention and all BA groups decreased IL-1β levels to varied degrees. When compared to the IR group, BA-H showed a mild improvement without statistical significance, but BA-L and WR-2721 considerably reduced IL-1β levels (*P* < 0.05). Additionally, both BA-H and WR-2721 therapies considerably raised SOD levels (*P* < 0.01) and dramatically decreased MDA levels (*P* < 0.05) in comparison to the IR group. Serum MDA and SOD levels in mice treated with BA-L showed some recovery, but there was no discernible statistical difference when compared to the IR group (**Figure 4A**). There was no discernible difference in the antibiotic intervention group BA (**Figure 4B**). Consistent with donor data, both normal microbiota and BA treatments enhanced oxidative stress and inflammatory responses to radiation-induced intestinal damage in FMT trials (**Figure 4C**). These results show that BA improves post-radiation antioxidant activity in the gut, lowers serum ROS levels in irradiated mice, and reduces radiation-induced intestinal inflammation in a microbiota-dependent manner.

### 3.5 BA alleviates intestinal barrier damage in RIII mice

**Figure 5:**
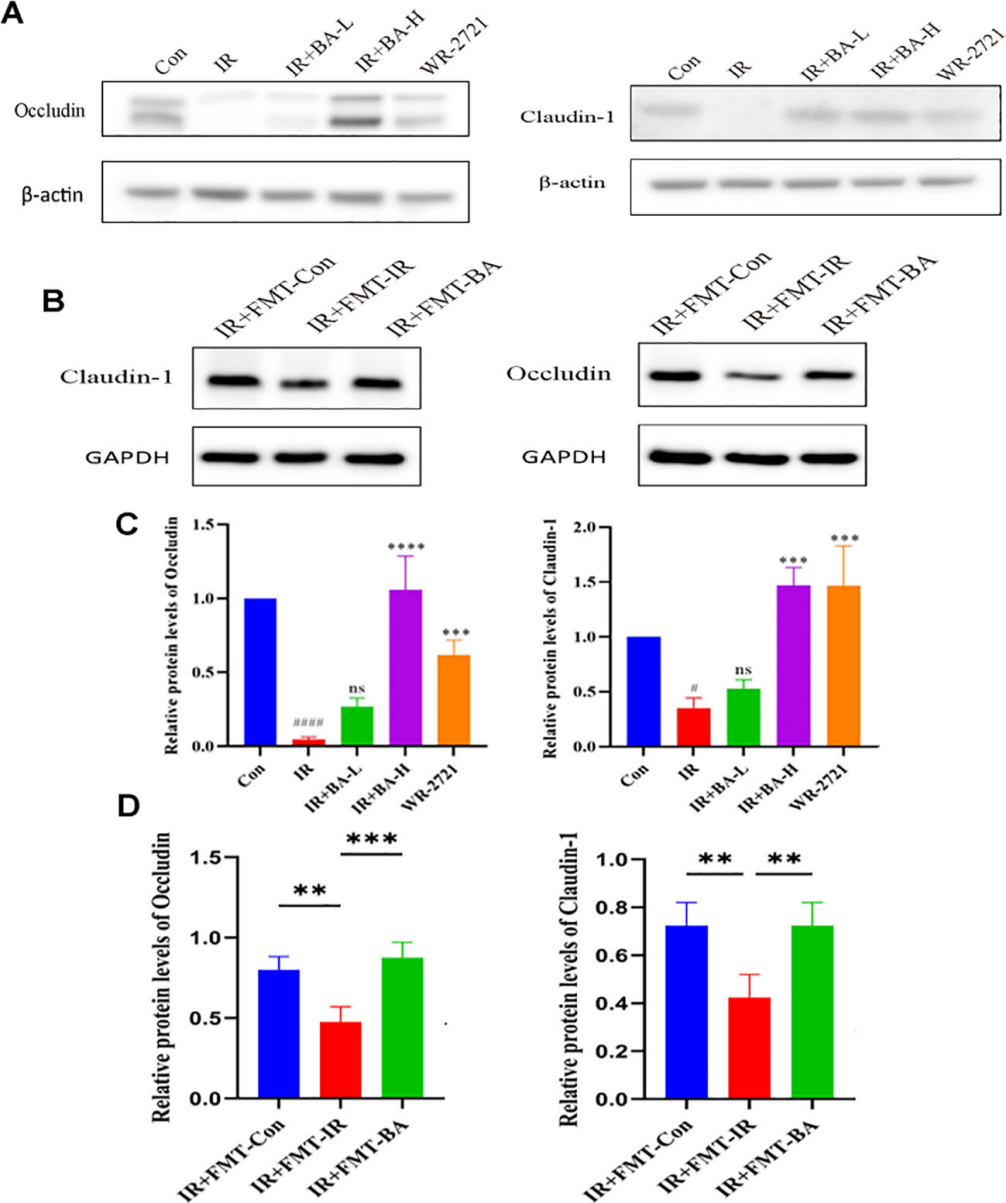
Effects of BA on gut barrier-related proteins in RIII mice (n=3). **(A)** Occludin and Claudin-1 protein bands in mice from each donor group; **(B)** Occludin and Claudin-1 protein bands in mice from each FMT group; **(C)** Quantitative analysis of protein band gray values in donor groups; **(D)** Quantitative analysis of protein band intensity in FMT groups; Compared with Con group, ^#^*P* < 0.05, ^####^*P* < 0.0001; Compared with IR group, **P < 0.01, ***P < 0.001, ****P < 0.0001, ns indicates no statistically significant difference.

Prior research has indicated that IR causes intestinal barrier disruption in animals. The intestinal barrier, which is vital for preserving the balance of the gut microbiota and reducing inflammation and intestinal immune responses, is largely composed of tight junctions (TJs) between intestinal epithelial cells (23). Claudin, occludin, and the ZO family are examples of intestinal TJ proteins that are important indicators of intestinal epithelial integrity and are essential for preserving barrier integrity and controlling permeability (24). TJ protein expression in mouse intestinal tissue was measured by Western blot analysis 3.5 days after irradiation in order to examine the impact of BA on the intestinal mucosal barrier in RIII mice. The IR group showed a significant decrease in the expression of the proteins occludin and Claudin-1. On the other hand, compared to the IR group, TJ protein expression was restored in both the BA and WR-2721 groups (*P* < 0.001). BA-H intervention had better impact on the expression of the proteins Claudin-1 and Occludin (**Figure 5A**). The IR+FMT-IR group showed a significant decrease in the expression of the proteins occludin and claudin-1. In both the FMT-BA and FMT-Con groups, TJ protein expression was restored (*P* < 0.01) (**Figure 5B**). Quantitative densitometric analysis of protein band intensities demonstrated that blueberry anthocyanin (BA) intervention significantly restored the expression levels of the tight junction proteins claudin-1 and occludin, which were downregulated by ionizing radiation (IR). This restoration indicates that BA alleviates radiation-induced intestinal barrier damage, at least in part, by repairing the compromised tight junction architecture (**Figures 5C, D**).

### 3.6 Effects of BA intervention on radiation-induced proliferation and survival of mouse intestinal stem cells

**Figure 6:**
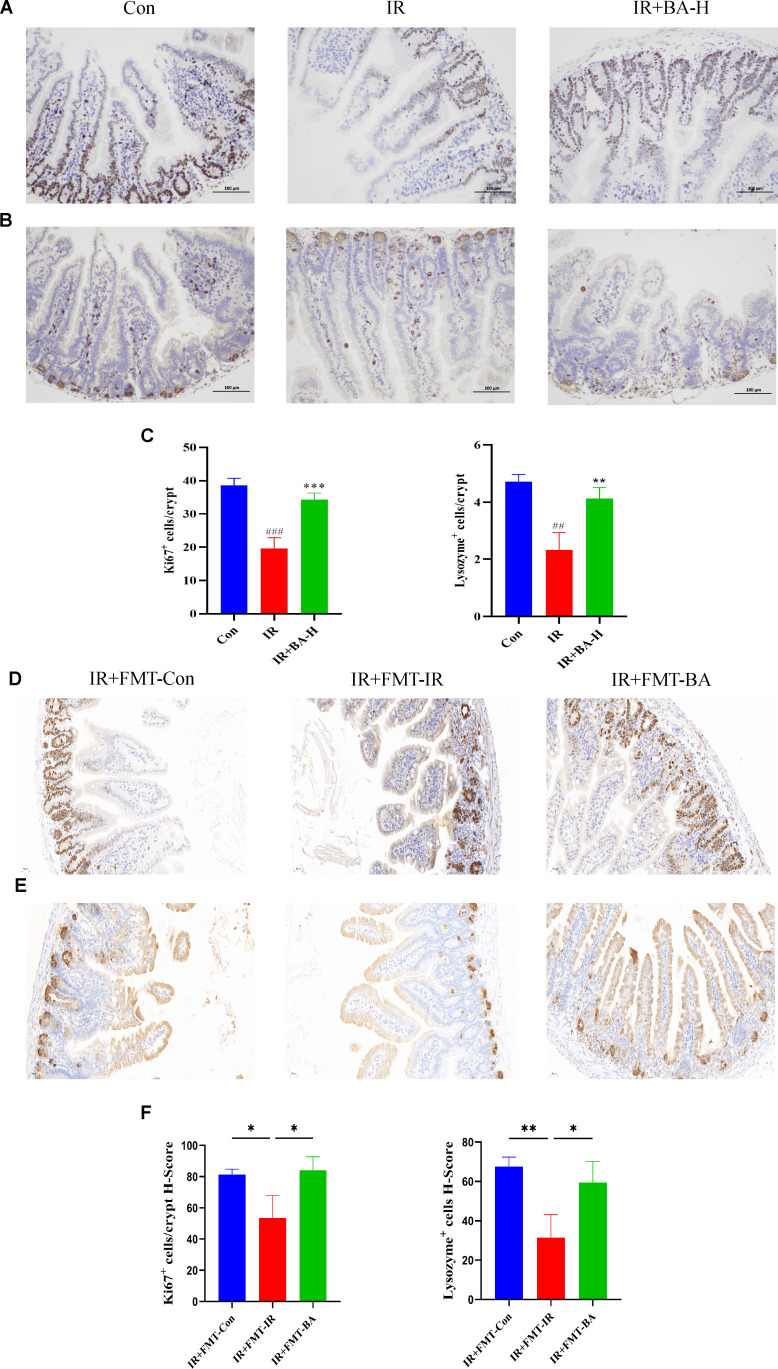
Effects of BA intervention on radiation-induced proliferation and survival of mouse intestinal stem cells (n=3). **(A)** Representative image of donor Ki67 immunohistochemical staining (200x); **(B)** Representative image of donor Lysozyme immunohistochemical staining (200x); **(C)** Quantitative analysis of cell numbers in donor crypts. Compared with the Con group, ^##^*P* < 0.01; compared with the IR group, **P < 0.01,**P<0.01; **(D)** Representative image of Ki67 immunohistochemical staining in the FMT group (200x); **(E)** Representative image of Lysozyme immunohistochemical staining (200x),; **(F)** Quantitative analysis of crypt cell numbers in the FMT group, *P < 0.05, **P < 0.01, ***P<0.001.

Every three to five days, small intestinal epithelial cells self-renew, and crypt stem cells are essential for intestinal regeneration after radiation damage. Research shows that 3.5 days after irradiation is when the growth of mouse intestinal crypt cells peaks (25). To evaluate Ki67^+^ proliferating cells in intestinal tissue, immunohistochemical examination was carried out 3.5 days after radiation. The mean quantity of Ki67^+^ cells in each crypt was measured. While the BA intervention dramatically increased Ki67^+^ cell counts (*P* < 0.001), the IR group showed a marked decrease in proliferating crypt cells (**Figure 6A**). Ki67^+^ cells significantly increased (*P* < 0.05) in both the IR+FMT-BA and IR+FMT-Con groups (**Figure 6D**). The Histochemistry Score, or H-score, is a histological grading technique used in immunohistochemistry processing. This score was utilized in the FMT experiment to assess how the BA intervention affected the survival and proliferation of radiation-induced mouse intestinal stem cells. A certain level of H-score rise (*P* < 0.01) was also observed in the BA group (**Figure 6C**). These findings suggest that BA facilitates the repair of injured intestinal tissue and increases the regeneration of intestinal crypt cells.

We looked into lysozyme^+^ Paneth cells in more detail. We measured changes in their numbers using lysozyme and Paneth cell immunohistochemical labeling. The average number of Lysozyme^+^ Paneth cells per crypt in the IR group was considerably lower at 3.5 days after radiation, according to the results, while the BA intervention restored positive cell counts (*P* < 0.01) (**Figure 6B**), which were close to normal levels. At the same time, the IR+FMT-BA group showed significantly higher H-scores (*P* < 0.05) (**Figure 6F**) and higher lysozyme^+^ Paneth cell counts (*P* < 0.05) (**Figure 6E**). All of these results suggest that BA intervention improves barrier function and encourages intestinal tissue healing in radiation-exposed animals.

### 3.7 BA treatment alters the diversity and composition of the gut microbiota in RIII mice

**Figure 7:**
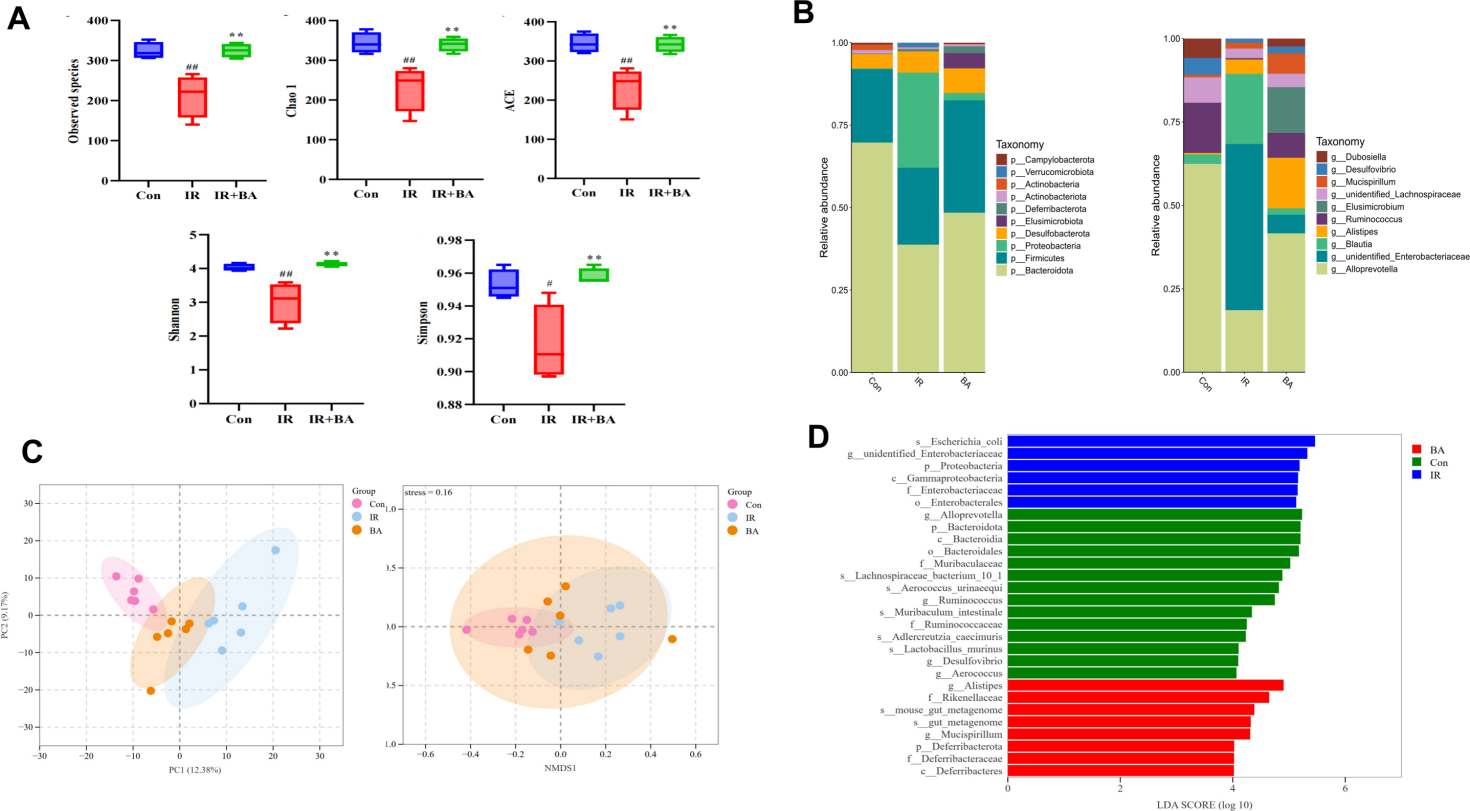
Effects of BA on gut microbiota diversity and composition in mice (n=6). **(A)** Effect of BA on α-diversity of gut microbiota in RIII mice, compared to the Con group ^#^*P* < 0.05, ^##^*P* < 0.01; compared to the IR group, **P < 0.01. **(B)** Effect of BA on gut microbiota structure in RIII mice **(C)** BA effects on RIII mouse gut microbiota β diversity **(D)** LDA value distribution bar chart.

We first examined the impact of BA on the α-diversity​ of the gut microbiota in irradiated mice. The species richness and evenness within each group were assessed using the Observed species, Chao1, ACE, Shannon, and Simpson indices. Compared to the Con group, the IR group showed a significant reduction in species richness (as indicated by lower Observed species, Chao1, and ACE indices) and diversity (lower Shannon and Simpson indices). BA intervention markedly mitigated these radiation-induced declines, restoring gut microbial α-diversity to a level comparable to the Con group (**Figure 7A**).

Next, to evaluate differences in microbial community structure between groups (β-diversity), we performed principal component analysis (PCA) and non-metric multidimensional scaling (NMDS). The results revealed distinct clustering of samples according to their groups. Samples within the same group clustered closely, indicating good intra-group reproducibility, while clear separations were observed between the Con, IR, and BA groups in both PCA and NMDS plots. This suggests that BA intervention effectively promoted a shift in the overall gut microbiota composition toward a state resembling that of the unirradiated controls (**Figure 7B**).

Species annotation results were utilized to create stacked bar charts that show the top 10 species by abundance at the phylum and genus levels across groups in order to better examine changes in the gut microbiota and make comparisons easier: *Bacteroidetes*, Fi*rmicutes, Proteobacteria, Desulfobacterota, Deferribacterota, Actinobacteriota*, and *Verrucomicrobiota* made up 99% of the mouse gut microbiota at the phylum level. With lower relative abundances of *Bacteroidetes* and *Actinobacteria* in the IR group and higher relative abundances of *Firmicutes, Proteobacteria*, and *Desulfobacterota*, indicating gut microbiota dysbiosis, radiation markedly changed the composition of the gut microbiota. The ability of BA to repair the structure of the mouse gut microbiota at the phylum level was demonstrated by the fact that the BA intervention raised the relative abundance of the Bacteroidetes phylum while lowering that of the *Proteobacteria* phylum in comparison to the IR group. *Alloprevotella*, *unidentified_Enterobacteriaceae, Blautia, Alistipes, Ruminococcus, unidentified_Lachnospiraceae*, and *Desulfovibrio* were the most prevalent genera of gut microbiota at the genus level. The relative abundances of *Alloprevotella, Ruminococcus, Lachnospiraceae, Dubosiella*, and *Desulfovibrio* were lower in the IR group than in the Con group, although the relative abundances of *Enterobacteriaceae* and *Blautia* were higher. The distribution of gut microbiota species was improved by BA intervention, which reversed radiation-induced alterations in relative abundance for *Prevotella, Ruminococcus, Lachnospiraceae, Desulfovibrio, Dubosiella, Bacteroides*, and *Enterobacteriaceae* (**Figure 7C**).

Biomarkers with statistically significant group differences are identified by LEfSe. Species with LDA > 4 and *P* < 0.05 were chosen based on statistical significance (*P*-value) and species contribution (LDA score). Fourteen taxa were found in the Con group, six in the IR group, and eight in the BA group at the five taxonomic levels (phylum, class, order, family, and genus). *Aerococcus urinaeequi, Ruminococcus, Muribaculum intestinale, Ruminococcaceae, Adlercreutzia caecimuris, Lactobacillus murinus, Aeromonas, Aerococcus, Prevotella, Bacteroidetes* phylum, *Bacteroidetes* class, *Bacteroidales* order, and *Muribaculaceae* family were among the enriched taxa in the Con group. *Escherichia coli*, *unidentified Enterobacteriaceae, Proteobacteria* phylum, *Gammaproteobacteria* class, *Enterobacteriaceae* family, and *Enterobacterales* order were among the enriched species in the IR group. *Alisterella*, *Rikenellaceae, Mucispirillum, Deferribacteres*, and *Deferribacteraceae* were all significantly enriched in the BA group (**Figure 7D**). The findings show that mice in the Con, IR, and BA groups had distinct gut microbiota species. It can be seen from the beneficial bacterial communities significantly enriched in the BA treatment group compared to the IR group that(**Table 1**), increasing probiotics and lowering pathogenic bacterial counts in the gut, BA helps mice with radiation-induced dysbiosis of the gut microbiota.

**Table 1:** Beneficial microbial communities significantly enriched in the BA treatment group (compared to the radiation group).

Genus name		Trends in the BA group (vs. the IR group)		Potential probiotic benefits	
*Ruminococcus*		Significant increase (↑)		Some strains produce SCFAs, which help regulate the immune system and maintain gut homeostasis.	
*Alloprevotella*		Significant increase (↑)		Produces SCFAs, provides energy to intestinal cells, reduces inflammation, and protects the intestinal barrier	
*Bacaeroides*		Significant increase (↑)		Some bacterial strains can produce succinic acid, which plays a role in immune regulation and is associated with gut homeostasis.	
*Lachnospiraceae*		Significant increase (↑)		Potentially beneficial bacteria that produce SCFAs are positively associated with anti-inflammatory effects and barrier function.	
*Desulfovibrio*		Significant increase (↑)		Participates in the intestinal sulfur cycle, metabolizes sulfate, and maintains microbial balance	
*Dubosiella*		Significant increase (↑)		Produces SCFAs, has anti-inflammatory effects, helps regulate the immune system, and inhibits intestinal inflammation	

↑ indicates an increase.

### 3.8 Effects of BA intervention on gut metabolites in RIII mice

**Figure 8:**
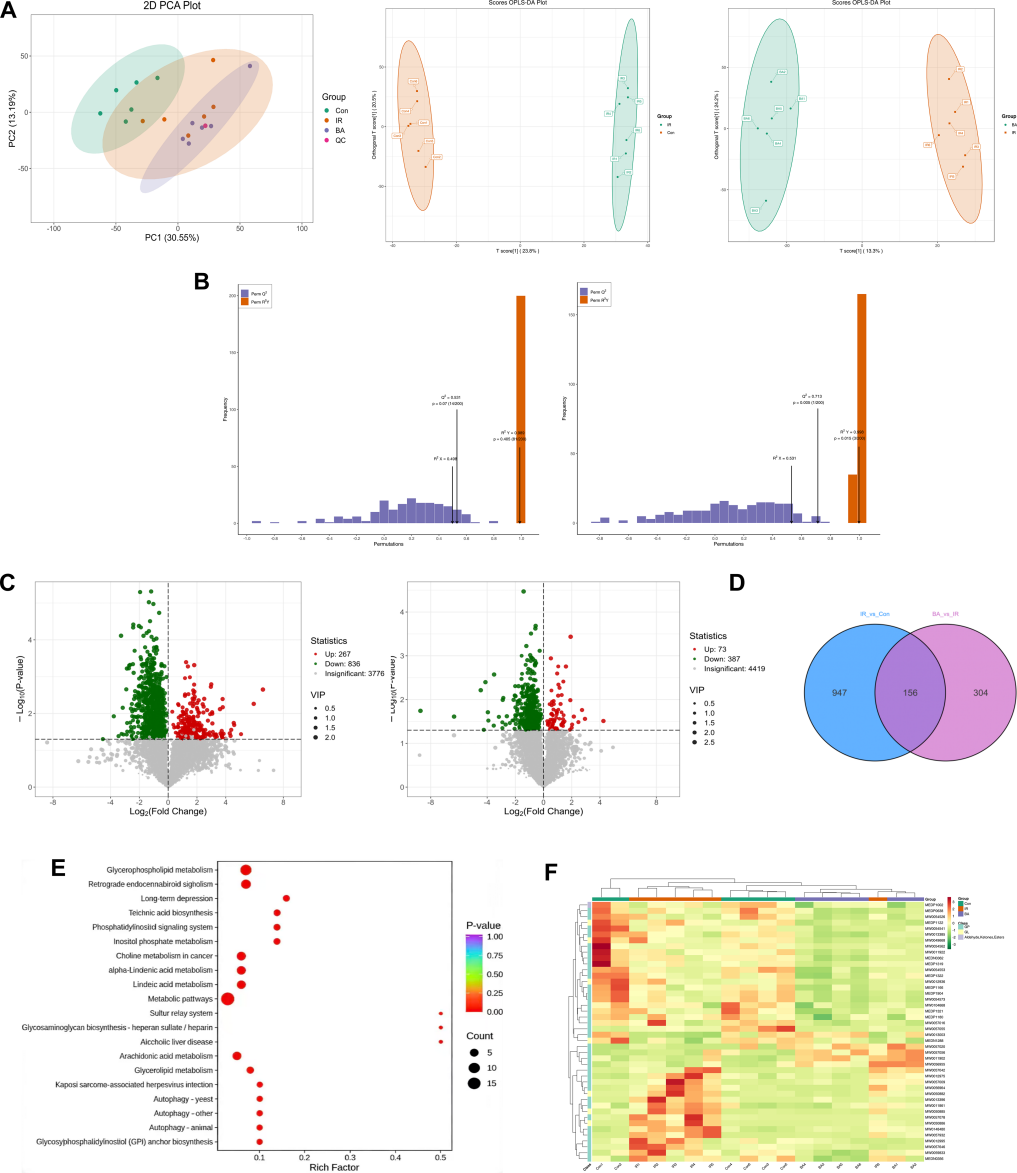
Effects of BA intervention on gut metabolites in RIII mice (n=6). **(A)** PCA and OPLS-DA score plots of mass spectrometry data for samples from each group and quality control samples; **(B)** Model evaluation; **(C)** Volcano plot of intergroup differential metabolites; **(D)** Venn diagram of differential metabolites across groups; **(E)** Pathway enrichment analysis of differential metabolites. **(F)** Heatmap of cluster analysis of gut metabolites in each group of mice.

This part further explores the protective mechanism of BA against RIII mice from a metabolomics perspective by using non-targeted metabolomics technology to examine the patterns of metabolite changes in the intestinal tissues of radiation-induced RIII mice. The findings show that PC2 accounts for 28.43% of the variance in raw data, whereas PC1 explains 12.12%. PC1 and PC2 exhibit distinct patterns of separation. QC samples show good system stability by clustering together with a much higher clustering intensity than other sample groups. However, the picture did not clearly depict classification patterns because of outlier data within groups. Consequently, additional PCA analysis between samples was carried out. PCA was used to compare all metabolites in the Con and BA groups with those in the IR group. With minimal outliers and good intra-group grouping, the data was balanced. Samples from the Con and IR groups, as well as those from the IR and BA groups, showed comparatively clear distinction. This suggests that mice’s metabolic patterns are altered by both BA intervention and RIII modeling. However, intergroup disparities were not as noticeable in PCA analysis because of individual differences and sampling errors. As a result, OPLS-DA was used once more to evaluate the model (**Figure 8A**). When comparing the Con and IR groups, the results showed that R2X = 0.531, R2Y = 0.998, and Q2 = 0.713. An effective model is indicated by a Q2 value greater than 0.5 (**Figure 8B**). The results of comparing the IR and BA groups were R2X = 0.498, R2Y = 0.989, and Q2 = 0.531; a valid model is indicated by Q2>0.5. There was a noticeable difference between the IR and BA groups, as well as between the Con and IR groups. As a result, the OPLS-DA model showed strong dependability, stability, and fitting. Metabolites in the Con, IR, and IR+BA groups were statistically compared using PCA and OPLS-DA analysis. *P* < 0.05 and VIP > 1 were used to identify metabolites as significantly different. The IR group exhibited substantial changes in 1,103 metabolites, including 267 downregulated and 836 upregulated metabolites, when compared to the Con group. In the IR+BA group, 460 metabolites were significantly different from the IR group; 387 metabolites showed a significant overexpression trend, whereas 73 metabolites showed a significant downregulation trend. The figure below provides a summary of the screening results(**Table 2**).

**Table 2:** Differential metabolite screening results.

Group name	All sig diff	Down regulated	Up regulated
Con vs IR	1103	267	836
IR vs BA	460	73	387

Volcano plots offer a graphic depiction of the statistical significance and variations in metabolite expression between two sample groups. In RIII mice, they each show distinct metabolic alterations brought on by IR and BA treatments (**Figure 8C**). Venn diagrams show the connections between metabolites that are expressed differently in various populations. The findings indicate that the Con vs. IR and IR vs. BA groups had 156 common differentially expressed metabolites (**Figure 8D**).

Glycerophospholipid metabolism, arachidonic acid metabolism, α-linolenic acid metabolism, linoleic acid metabolism, phosphatidylinositol signaling, and glycosylphosphatidylinositol anchor biosynthesis were the main pathways involved in radiation-induced metabolic disorders in the mouse small intestine, according to KEGG pathway enrichment analysis based on the differential metabolite results. Metabolite levels were reduced, and associated metabolic circuit disruption was enhanced in the BA group, confirming metabolically that BA has a positive restorative effect on RIII animals. Concurrently, the heatmap showed possible biomarker clustering patterns. The Con and IR groups showed significant variations in biomarker levels, whereas the BA group’s levels were different from the IR group and more similar to the Con group (**Figure 8E**).

## 4 Discussion

The protective benefits of blueberry anthocyanins (BA) against radiation-induced intestinal injury (RIII) and its possible gut microbiota-dependent mechanisms were methodically examined in this study. In mice exposed to RIII, our results show that BA intervention significantly increased survival rates, reduced weight loss, reduced intestinal histopathological damage, inhibited oxidative stress and inflammatory responses, and encouraged intestinal barrier repair and epithelial cell regeneration. Importantly, we verified that these positive benefits of BA mostly rely on its regulatory influence on the gut microbiome by fecal microbiota transplantation (FMT) validation and antibiotic-induced microbiota depletion tests.

Our results are in line with an increasing amount of preclinical research that suggests dietary polyphenols may have radioprotective benefits via altering the gut flora, even if there are currently no clinical studies on the use of BA for the treatment of RIII. For instance, it has been demonstrated that the carotenoid polyphenol lycopene reduces radiation-induced intestinal damage in rats, an effect linked to enhanced Lactobacillus abundance and the restoration of microbial diversity (26). Similarly, by preserving intestinal stem cell function, grape seed procyanidin extract can reduce radiation-induced intestinal damage; this effect is also linked to positive modifications in the gut microbiota (27). Furthermore, epigallocatechin gallate’s capacity to alter the gut microbiota, boost the number of butyrate-producing bacteria, and consequently lower inflammation and oxidative stress is directly related to its protective effects against radiation-induced intestinal and hematopoietic damage (28). The mode of action of BA in this study clearly shares mechanistic similarities with the previously mentioned polyphenolic compounds. Specifically, BA functions as a dietary modulator of the microbiota to shape a gut microenvironment that is favorable to maintaining metabolic homeostasis, anti-inflammatory balance, and epithelial integrity, thereby exerting radioprotective effects.

The degree of radiation-induced intestinal injury is largely influenced by changes in the gut microbiota and their metabolites, which are brought on by IR (29). The pathophysiology of RIII is significantly influenced by gut dysbiosis, as previous research has shown that radiation alters the diversity, structure, and function of the gut microbiota (30). 16S rDNA sequencing technology has been the go-to technique for researching microbial communities due to its constant improvement.

RIII has a complicated pathophysiological mechanism that includes a number of factors, including intestinal mucosal damage (31) vascular endothelial damage (32) and dysbiosis (33) Although the exact mechanism is still unknown, the natural plant extract anthocyanin shows protective properties against RIII. Through non-targeted examination of endogenous metabolite alterations, metabolomics—a systems biology approach—allows for a thorough knowledge of physiological and pathological states (34). As the byproducts of cellular activity that represent intricate biochemical processes, metabolites are vital to human health and well-being (35). In order to detect and assess the effects of BA on intestinal tissue metabolism in RIII mice, this study used metabolomics, which revealed the drug’s multi-pathway, multi-target mode of action.

In this project, the structure and composition of the mouse gut microbiota were examined using 16S rDNA sequencing. Decreases in gut microbial diversity (Shannon and Simpson indices) and richness (Observed Species Index, Chao 1 Index, and ACE Index) were indicators of post-radiation dysbiosis, according to the results. whereas the BA intervention brought α diversity back to levels comparable to those of the control group. The gut microbial structure and composition of the Con and BA groups differed significantly from that of the IR group, according to PCA and NMDS analysis of the microbial community composition across groups. This implies that BA intervention has a beneficial effect on RIII mice’s gut microbiota healing.

The BA intervention dramatically decreased dangerous bacteria such as Enterobacteriaceae, hence reducing radiation-induced intestinal inflammation, according to LEfSe analysis (LDA > 4), which revealed statistically different microorganisms between groups. In conclusion, radiation upset the mouse gut microbiota’s equilibrium, whereas BA treatment increased the variety and quantity of microorganisms. This was accomplished by reducing opportunistic pathogens and raising the percentage of beneficial bacteria, which corrected radiation-induced dysbiosis and reduced RIII.

Additionally, radiation increased the relative abundance of *Firmicutes, Proteobacteria*, and *Desulfovibrionites* in mouse intestines while decreasing that of *Bacteroidetes* and *Actinobacteria* at the phylum level. In line with earlier research, the relative abundance of *Bacteroidetes* increased after BA intervention, although *Proteobacteria* declined (36). *Proteobacteria* make lipopolysaccharides (LPS), and high levels of LPS can kill macrophages and make inflammatory cytokines (37). Additionally, research shows that gastrointestinal inflammation is linked to higher *Proteobacteria* abundance (38). This suggests that BA intervention aids in mitigating the dysbiosis of the gut microbiota brought on by radiation. The health of the gut microbiota is reflected in the *Bacteroidetes/Firmicutes* ratio. Although there was no discernible change following the BA intervention, this ratio declined in the IR group relative to the Con group in our investigation, suggesting that mice in the BA group continued to show some degree of gut microbiota imbalance following radiation exposure. Acetate and propionate, two crucial short-chain fatty acids in the gut, are produced by Bacteroidetes, according to research (39), In addition to giving intestinal epithelial cells energy, it also directly stimulates the expression of tight junction proteins (such as occludin and claudin-1) and has anti-inflammatory effects by suppressing histone deacetylases. This provides a potential metabolic explanation for the reduced inflammation and intestinal barrier repair in Group BA. Both the immunological response and the gut mucosal barrier depend on this.

At the genus level, radiation exposure increased the relative abundance of *Enterobacter* and *Bacteroides* in mouse intestines while decreasing that of *Prevotella*, *Ruminococcus, Spirochaeta, Dubosella* and *Desulfovibrio*. BA intervention enhanced the species range of these genera and restored their relative abundance. *Prevotella* is a genus that is often linked to plant-based diets because it can break down foods that contain protein and carbohydrates, functions as a probiotic in the body (40), and increases the production of short-chain fatty acids, which increases intestinal tissue resistance to immunological stimulation (41). The primary cause of fiber degradation in the digestive tract is *Ruminococcus*, which also contributes to the formation of the intestinal microbial barrier and produces a variety of metabolites (42). The genus *Clostridium* is a significant butyrate-producing bacterium that interacts with intestinal microbes and takes part in human metabolism in the gut. As the colonic epithelium’s main energy source, butyrate also reduces inflammation and provides gut microorganisms energy, which encourages the proliferation of epithelial cells. Moreover, intestinal tight junction (TJ) protein expression is intimately linked to butyrate synthesis. According to research, the beneficial bacterial group *Dubosellaceae* is known to produce short-chain fatty acids and can lower systemic inflammatory responses (43). Its mechanism for radiation-induced intestinal damage is yet unknown, though, and it needs more research. Hydrogen sulfide is produced by Vibrio desulfurizans, which increases intestinal permeability. After BA intervention, there were no notable alterations. *Enterobacteriaceae* are linked to chronic intestinal inflammation because of their propensity for pathogenicity and rapid colonization (44). Intestinal inflammation brought on by radiation decreases butyrate-producing bacteria, which raises nitrate production and encourages *Enterobacteriaceae* growth (45). Because of its potential to improve host energy metabolism and reduce inflammation, the anaerobic Gram-positive species Brachybacteria, which is frequently found in mammalian gastrointestinal tracts, has attracted a lot of attention (46).

To examine the protective effects of BA on RIII mice, this work used the RIII mouse model to perform a non-targeted metabolomics investigation of colonic metabolites. Overlay analysis of total ion chromatograms from intestinal QC samples was used to verify the instrument’s stability and the accuracy of the experimental findings. Changes in metabolic patterns between the Con and IR groups, as well as between the IR and BA groups, were shown by analysis techniques such as OPLS-DA and volcano plots. Glycerophospholipid metabolism, arachidonic acid metabolism, α-linolenic acid metabolism, linoleic acid metabolism, phosphatidylinositol signaling, and glycosylphosphatidylinositol anchor production were the main pathways linked to radiation-induced metabolic dysregulation.

By increasing GPL metabolism, damage to glycerophospholipid biomembranes may cause inflammation. Then, by altering the production of lipoprotein lipase, inflammatory cytokines disrupt GPL metabolism (47). Moreover, inflammatory mediators intensify lipid oxidation, exacerbating the inflammatory condition. Radiation exposure changes phospholipase activity, modifies the phosphatidylcholine (PC) content, and causes functional problems and systemic inflammation (48). Thus, bile acids (BAs) are thought to preserve intestinal cell integrity by inhibiting radiation-induced cellular damage via PC control.

Oxidative stress can be regulated in both directions via arachidonic acid metabolism. Proinflammatory markers such as serum IL-1β and TNF-α dramatically rose in mice after radiation exposure. In addition to acting as a substrate for the production of inflammatory mediators implicated in inflammatory responses, these proinflammatory factors cause PC and PE to release arachidonic acid (49). Studies show that α-linolenic acid reduces and inhibits inflammation, but linoleic acid promotes inflammatory responses, which is a critical function of linoleic acid in immune modulation (50). It is thought that mice’s post-radiation oxidative stress and inflammatory reactions cause PC to produce more linoleic acid, which in turn causes pro-inflammatory reactions (51). A key player in phosphatidylinositol signaling, phosphatidylinositol 3-kinase (PI3K) regulates cellular development, proliferation, and metabolism. The PI3K/Akt signaling pathway suppresses IL-10 while increasing the production of TNF-α, IL-6, IL-8, and other cytokines (52). Therefore, the phosphatidylinositol signaling system’s activation, which intensifies inflammatory responses, may be linked to the development of RIII. Additionally, research suggests that GPI-Anchor can stimulate the immune system and support healthy gut flora (53). Furthermore, intestinal epithelial cell polarity, which is essential for the formation of the intestinal barrier and is critical for gut health, may be partially influenced by GPI-Anchor (54).

According to the results of this study, blueberry anthocyanins (BA) mitigated radiation-induced intestinal damage while concurrently changing the structure of the gut microbiota and restoring the host’s colonic metabolic profile. Beneficial bacterial groups like the Bacteroidetes phylum, Bacteroides, and Ruminococcus, which are important producers of short-chain fatty acids (such as acetic acid and propionic acid), were considerably enhanced by BA intervention. In addition to directly supplying energy to intestinal epithelial cells and improving intestinal barrier function by increasing the expression of tight junction proteins, these metabolites derived from the microbiota may also have anti-inflammatory effects by inhibiting histone deacetylases. The decreased inflammation and intestinal barrier repair seen in the BA-treated group are compatible with these processes. Nevertheless One limitation of this study is the absence of a blueberry anthocyanin (BA)-only control group (without irradiation). While previous studies have confirmed the safety and negligible physiological effects of BA at the doses used (50–200 mg/kg bw) in healthy rodents, inclusion of a BA-only group would have allowed for a more rigorous assessment of baseline microbial and metabolic alterations independent of radiation. Future studies should consider incorporating this group to fully elucidate the standalone effects of BA.”

Simultaneously, the gut microbiota controls the balance between the substrates and products of the lipid metabolic pathways that BA modulates, such as linoleic acid and arachidonic acid (eicosanoids). We speculate that, by metabolizing dietary fatty acids, the BA-modulated microbiota may increase the synthesis of anti-inflammatory mediators (like conjugated linoleic acid) and decrease the synthesis of pro-inflammatory eicosanoids (like PGE2), thereby synergistically exerting anti-radiation damage effects at the metabolic level. Additionally, the phosphatidylinositol signaling pathway and glycerophospholipid metabolism are upregulated, indicating that BA intervention aids in stabilizing the biological structure of intestinal epithelial cells. Cell survival, proliferation, and signal transmission all depend on the integrity of the cell membrane. It has been demonstrated that microbial metabolites, such as short-chain fatty acids, affect the lipid metabolism of host cells. Therefore, through its metabolites, the BA-modulated microbiota may indirectly affect the host’s membrane lipid composition and associated signaling pathways (such as PI3K/Akt), encouraging intestinal epithelial cell proliferation and boosting resistance to radiation damage. Future research should use integrated metagenomic-metabolomic analysis, germ-free animal models, and particular metabolite supplementation experiments to directly test this “microbiota-metabolite-host” interaction network.

This study established the crucial involvement of the microbiota through antibiotic depletion and FMT tests, and it used 16S rDNA sequencing and non-targeted metabolomics to identify synergistic changes in microbial community structure and metabolic profiles following BA intervention. However, single-bacterial colonization studies, metabolite supplementation studies, or germ-free animal models are still needed to validate which particular beneficial bacteria and their metabolites act as the direct effector molecules mediating the protective effects of BA. While metabolomic pathway enrichment analyses (e.g., glycerophospholipid metabolism, arachidonic acid metabolism) offer valuable insights into the mechanism of action of BA, the molecular mechanisms by which microbial metabolites regulate particular signaling targets (e.g., key enzymes, receptors) remain unclear. To do multi-omics analysis, future research should combine transcriptomics, proteomics, and gene knockout methods.

In conclusion, this study shows that BA intervention improves radiation-induced gut microbial dysbiosis, preserves intestinal barrier integrity, and efficiently regulates intestinal metabolites in RIII mice. By controlling several metabolic pathways, such as glycerophospholipid metabolism, arachidonic acid metabolism, linoleic acid and α-linolenic acid metabolism, the phosphatidylinositol signaling system, and GPI anchor biosynthesis, BA may shield the intestine from radiation damage. A promising new approach to treating and preventing RIII is provided by BA intervention. Its mode of action hasn’t been thoroughly investigated in this study, though. In order to provide more thorough scientific proof for the clinical use of BA in RIII prevention and treatment, future studies should make use of multi-omics technologies to methodically examine and evaluate its action pathways.

## 5 Conclusion

This work demonstrates that blueberry anthocyanins (BA) ameliorate radiation-induced intestinal injury (RIII) by remodeling the gut microbiota and associated host metabolism. 16S rDNA sequencing revealed that BA enriched beneficial taxa, such as *Bacteroidetes, Ruminococcus*, and *Lachnospiraceae*. Critically, antibiotic-induced microbiota depletion abolished the protective effects of BA, whereas fecal microbiota transplantation (FMT) from BA-treated donors recapitulated these benefits, establishing the gut microbiota as a necessary mediator of BA’s action. Non-targeted metabolomics further showed that BA significantly reversed radiation-induced dysregulation of metabolic pathways, including glycerophospholipid and arachidonic acid metabolism. The amelioration of oxidative stress and inflammation was associated with the enrichment of short-chain fatty acid-producing bacteria, suggesting microbiota-driven metabolic reprogramming. Concurrently, BA intervention directly enhanced​ intestinal barrier repair, as evidenced by upregulated tight junction proteins (occludin, claudin-1), increased intestinal stem cell proliferation (Ki67^+^), and improved Paneth cell survival (lysozyme^+^). This barrier repair is likely facilitated by metabolites derived from the BA-modulated microbiota.​Collectively, these findings define a functional “BA–Gut Microbiota–Metabolite–Intestinal Barrier” axis, positioning BA as a promising microbiota-targeting agent for the prevention of RIII. Future studies should focus on elucidating the specific roles of the key beneficial bacterial clusters enriched by BA and the bioactive metabolites they produce, to fully delineate the mechanistic cascade.

## Data Availability

The original contributions presented in the study are publicly available. This data can be found here: [MTBLS14609, https://www.ebi.ac.uk/metabolights/MTBLS14609].
